# Safety and Efficacy of Stroke Thrombolysis for Patients with Cerebral Cavernous Malformations: Literature Review and Nationwide Cohort Study

**DOI:** 10.3390/neurosci7010024

**Published:** 2026-02-08

**Authors:** Huanwen Chen, Rachel K. Laursen, Matthew K. McIntyre, Monika Jain, Hamza A. Salim, Dhairya A. Lakhani, Ajay Malhotra, Dheeraj Gandhi, Marco Colasurdo

**Affiliations:** 1 Neurosurgery, University of Maryland Medical Center, Baltimore, MD 21201, USA; alvin.huanwen.chen@gmail.com (H.C.);; 2 Neurology, Oregon Health and Sciences University, Portland, OR 97239, USA; 3 Neurosurgery, Oregon Health and Sciences University, Portland, OR 97239, USA; 4 Neurology, University of Maryland Medical Center, Baltimore, MD 21201, USA; 5 Neuroradiology, MD Anderson Cancer Center, Houston, TX 77030, USA; 6 Neuroradiology, West Virginia University, Morgantown, WV 26506, USA; 7 Radiology, Yale New Haven Hospital, New Haven, CT 06510, USA; 8 Interventional Radiology, Oregon Health and Sciences University, Portland, OR 97239, USA

**Keywords:** stroke, infarct, thrombolysis, cavernoma, cavernous malformation, hemangioma, hemorrhage

## Abstract

Background: Intravenous thrombolysis (IVT) is relatively contraindicated in acute ischemic stroke (AIS) patients with intracranial vascular malformations per current guidelines. Thus, the presence of cerebral cavernous malformations (CCMs) may complicate treatment decision-making. Methods: We performed a literature review of the PubMed, Embase, Scopus, and Web of Science databases through July 2025, identifying reported cases of IVT administration in AIS patients with CCMs. Additionally, we conducted a retrospective cohort study using the Nationwide Readmissions Database (2016–2022) of AIS patients with CCM, and assessed outcomes with IVT versus no IVT treatment. The primary outcome was functional independence at discharge; secondary outcomes included mortality and intracranial hemorrhage (ICH). Results: Only 34 CCM patients across 7 studies were identified in the literature, with symptomatic ICH occurring in 2 cases (5.9%). In the nationwide cohort, 846 AIS patients with CCMs were included, of whom 240 (28.4%) received IVT. Compared to no IVT treatment, IVT was associated with significantly higher rates of functional independence (46.4% vs. 24.6%, adjusted OR [aOR] 3.04 [95% CI 1.98–4.68], *p* < 0.001), without significant differences in mortality (8.5% vs. 8.3%, aOR 1.40 [95% CI 0.52–3.76], *p* = 0.50) or ICH (20.3% vs. 16.1%, adjusted OR 1.01 [95% CI 0.53–1.93], *p* = 0.97). Conclusions: The current literature on the safety and efficacy of IVT in AIS patients with CCMs is limited. Our nationwide study suggests that IVT was associated with higher rates of early functional independence without increased risks of hemorrhage or death among patients with CCM.

## 1. Introduction

Cerebral cavernous malformations (CCMs) are low-flow vascular lesions that affect approximately 0.1–0.5% of the general population, with annual bleeding rates ranging from 0.3% to 2.8% per year [1,2,3]. The presence of CCMs in patients with acute ischemic stroke creates significant clinical uncertainty regarding the safety and efficacy of intravenous thrombolysis (IVT). The current guidelines list the presence of vascular malformations as a relative contraindication to IVT due to theoretical hemorrhage risk; however, limited evidence exists to inform clinical decision-making, specifically for patients with CCMs [4]. The rarity of this clinical scenario has precluded the conduct of adequately powered studies, leaving clinicians to make treatment decisions based on small case series and theoretical considerations.

In this report, we performed a comprehensive review of the current literature on the safety and efficacy of IVT in AIS patients with CCMs. We also queried the Nationwide Readmissions Database (NRD), a nationally representative database of hospitalization records in the United States, to assess real-world outcomes of IVT in AIS patients with CCMs.

## 2. Methods

### 2.1. Literature Search and Review

We searched the PubMed, Embase, Scopus, and Web of Science databases from inception up to July 2025 for English-language clinical articles reporting outcomes following the use of IVT for AIS patients with CCM. The search strategies for each database are detailed in Table S1. Original articles, letters, and conference abstracts (including case reports, case series, cohort studies, and clinical trials) that describe the outcomes of IVT treatment for stroke patients with CCMs were included. Review articles and duplicate reports were excluded. The titles and abstracts of the search results were screened independently by two investigators for eligibility. Subsequently, full-text screening was also conducted by two separate investigators.

### 2.2. Nationwide Readmissions Database

For the NRD portion of our study, we conducted a retrospective cohort study that utilized the Healthcare Cost and Utilization Project (HCUP) Nationwide Readmissions Database (NRD) from 2016 to 2022 using an established study design for comparative effectiveness [5,6,7]. The NRD contains data from approximately 18 million annual discharges across 30 states, representing the largest publicly available all-payer inpatient database in the United States. Since the NRD does not contain patient identifiers and is retrospectively queried, this study was exempt from institutional review board approval and informed consent.

### 2.3. Study Population

We identified adult patients with moderate to severe AIS (defined as NIHSS > 4) and a concurrent diagnosis of CCM using ICD-10 diagnostic codes (Table S2). Patients with comorbidities known to significantly increase bleeding risk that may contraindicate intravenous thrombolysis under current treatment guidelines—including endocarditis, vasculitis/arteritis, cerebral amyloid angiopathy, moyamoya disease, cancer, intracranial tumor, or other cerebrovascular malformations—were excluded [8].

### 2.4. Intervention and Outcomes

The primary exposure was treatment with IVT. The primary efficacy outcome was functional independence at hospital discharge, defined as routine discharge to home without rehabilitation needs, which has been used as a surrogate marker for excellent neurological outcomes in prior NRD studies of AIS patients [9,10,11]. Secondary outcomes included in-hospital mortality, any ICH, subarachnoid hemorrhage, intraparenchymal hemorrhage, and hospital length of stay. All outcomes are assessed as occurrences within or at the end of hospitalization events.

### 2.5. Other Variables

Patient age, demographics, and stroke severity were captured. Comorbidities such as atrial fibrillation, hypertension, hyperlipidemia, intracranial atherosclerosis, smoking history, and others were also recorded (full list in Table 2). An Elixhauser comorbidity index was calculated for each patient to quantify the overall comorbidity burden [12].

### 2.6. Statistical Analysis

Descriptive data were presented as median (Q1–Q3) and percentages, with comparisons performed using non-parametric tests or chi-squared tests. Discharge weights were applied to all patient counts per standard HCUP protocol to generate nationally representative sample sizes. All statistical comparisons accounted for survey weights. Multivariable logistic regression models were used to assess associations between IVT and binary outcomes. Poisson regressions were used to analyze length of stay. All variables from Table 2 were included for adjustment purposes. Given that endovascular thrombectomy (EVT) may be independently associated with ICH unrelated to CCMs, we performed a sensitivity analysis investigating the safety and efficacy of IVT among AIS patients who did not undergo thrombectomy. Additionally, given that baseline coagulopathy anticoagulation use may also modulate treatment decisions and hemorrhagic risk in the non-treated group, we also performed sensitivity analyses excluding these patients. The results were presented as odds ratios or rate ratios with 95% confidence intervals, with statistical significance defined as *p* < 0.05.

## 3. Results

### 3.1. Literature Review

The literature review yielded 95 entries—34 from Embase, 29 from Scopus, 19 from Web of Science, and 13 from PubMed. After removing duplicates, 56 entries were screened for eligibility, 9 underwent full-text review, and ultimately, 7 studies were included [13,14,15,16,17,18,19]. Overall, 34 CCM patients were identified; symptomatic ICH occurred in 2 cases (5.9%, Table 1). In a single study comparing post-IVT outcomes in 9 CCM and 341 non-CCM patients, Erdur et al. found no difference in rates of symptomatic hemorrhage between the two groups (11.1% vs. 7.9%, *p* = 0.27). Due to small sample sizes and the predominance of case reports and series, meta-analysis was not performed.

### 3.2. NRD Study Population

**Figure 1:**
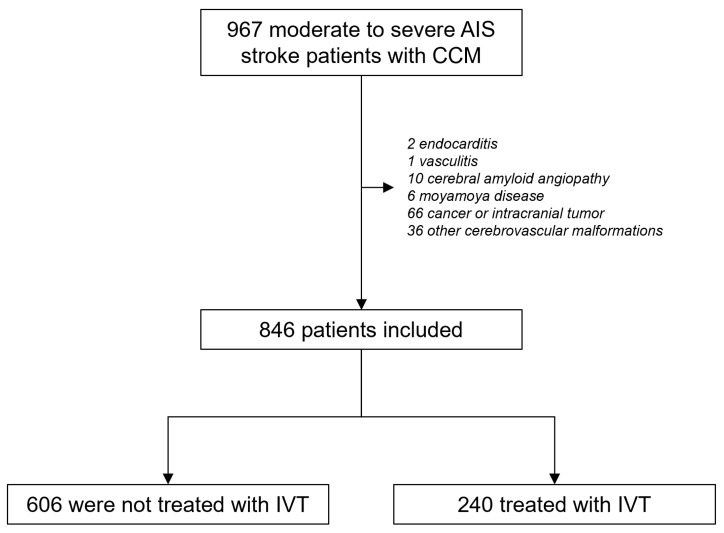
Study flow chart. Abbreviations: AIS—acute ischemic stroke, CCM—cerebral cavernous malformations, IVT—intravenous thrombolysis.

Next, we queried the NRD for cases of CCM patients hospitalized for AIS. Among 966 patients with CCMs hospitalized for moderate to severe AIS, 846 were included in the final analysis after applying exclusions (Figure 1). Of the included patients, 240 (28.4%) received IVT, while 606 (71.7%) did not receive IVT. The study cohort had a median age of 71 years, was predominantly male (53.2%), and most patients (85.8%) were treated at urban teaching hospitals.

### 3.3. Patient Characteristics

Table 2 presents the baseline characteristics of the study population. Patients who received IVT were significantly younger than those who did not (median age 69 versus 72 years, *p* = 0.021). The median NIHSS score was 9 in both treatment groups, indicating similar stroke severity. As expected, patients in the no-IVT group had significantly higher rates of chronic anticoagulant use (9.9% versus 3.6%, *p* = 0.010). Patients who received IVT had a significantly higher prevalence of headache disorders (10.9% versus 4.5%, *p* = 0.012). Of note, given the number of characteristics compared, interpretation of these significant baseline differences require caution due to high risks of false discovery with multiple comparisons. No significant differences were observed between groups regarding sex distribution, hospital type, stroke severity, rates of EVT, or other comorbidities. The Elixhauser comorbidity index was similar between groups, with a median score of 12.

### 3.4. Clinical Outcomes

Table 3 presents the primary and secondary outcomes. The rate of functional independence at the time of hospital discharge was significantly higher among patients who received IVT compared to those who did not (46.4% versus 24.6%, *p* < 0.001), representing a number needed to treat of 4.6. After multivariable adjustment, IVT was associated with a three-fold increase in the odds of achieving functional independence (OR 3.04, 95% CI 1.98–4.68, *p* < 0.001), representing a 3-fold increase.

Regarding safety outcomes, mortality rates were similar between groups (8.5% for IVT versus 8.3% for no-IVT, *p* = 0.961), with no significant difference after adjustment (OR 1.40, 95% CI 0.52–3.76, *p* = 0.50). Hemorrhagic complications showed no significant differences between treatment groups. Any ICH occurred in 20.3% versus 16.1% (*p* = 0.30), with intraparenchymal hemorrhage occurring in 18.2% versus 13.3% (*p* = 0.22) and subarachnoid hemorrhage in 3.5% versus 3.3% (*p* = 0.93). After multivariable adjustment, IVT was not associated with a significantly increased risk of hemorrhagic outcomes in AIS patients with CCM.

Patients who received IVT had significantly shorter hospital stays with a median length of 4 days compared to 6 days for those who did not receive IVT (*p* = 0.014). The adjusted analysis showed a 35% reduction in length of stay associated with IVT treatment (Poisson rate ratio 0.65, 95% CI 0.53–0.81, *p* < 0.001).

### 3.5. Sensitivity Analyses

Given that some patients in our cohort also underwent EVT, which may influence hemorrhage risk independent of CCMs, we performed a sensitivity analysis excluding those who underwent EVT. Here, the results were largely similar, with IVT showing significantly higher rates of functional independence, without higher risk of mortality or ICH (Table S3).

Finally, given that prior use of anticoagulation or pre-existing coagulopathy may simultaneously contraindicate IVT use and modulate bleeding risks, we performed a final sensitivity analysis excluding these patients. Here, IVT remained significantly associated with higher rates of functional independence and no significant increase in the rates of ICH (Table 4).

## 4. Discussion

In this study, we performed a comprehensive review of the current literature on the safety and efficacy of IVT for CCM patients presenting with AIS. To date, only 34 cases have been reported, with 2 cases of symptomatic ICH, representing 5.9% of reported cases. Only one study has compared the safety of IVT in CCM patients to non-CCM patients, reporting no significant difference in hemorrhage rates; however, the results were severely limited by a small sample size with only nine CCM patients. To address this knowledge gap, we performed a large-scale, nationwide analysis of AIS patients with CCMs in the United States. Among 846 CCM patients hospitalized with AIS, 240 received IVT. IVT was significantly associated with a three-fold increase in the odds of functional independence (number needed to treat of 4.6, an effect size consistent with prior clinical trials of IVT in the general population [20,21]), without an increased risk of in-hospital mortality or hemorrhagic complications. This study represents the largest cohort study to date evaluating the use of IVT in AIS patients with CCMs, and it provides reassurance that IVT may be associated be higher rates of early functional independence without increasing risks of hemorrhage and death.

The relatively low added risk of hemorrhage following IVT in patients with CCMs—especially in comparison to other cerebrovascular malformations such as large aneurysms and arteriovenous malformations (AVMs) [22,23]—may be attributable to distinct vascular architecture and hemodynamic features. CCMs consist of low-flow, capillary-like vessels that lack high-pressure arterial inflow, possibly making CCMs less prone to thrombolysis-induced rupture. In contrast, vascular lesions with high-flow dynamics, such as AVMs and aneurysms, may be more prone to thrombolysis-induced rupture, particularly in the setting of AIS, where blood pressure is often elevated in response to acute ischemia [24]. Furthermore, even in the event of rupture, the low-flow nature of CCMs typically results in small-volume bleeds that are less likely to cause mass effect, neurological deterioration, or require surgical intervention. Many hemorrhages associated with CCMs may be intracapsular, which may not meaningfully affect the surrounding tissue. As such, hemorrhagic events associated with CCMs are often clinically milder than those arising from other cerebrovascular lesions, with only a 2.2% mortality risk associated with rupture events [2]. Although IVT may increase the risk of CCM rupture, the substantial benefits of timely reperfusion in AIS likely outweigh the risks associated with the generally benign clinical course of CCM rupture.

**Figure 2:**
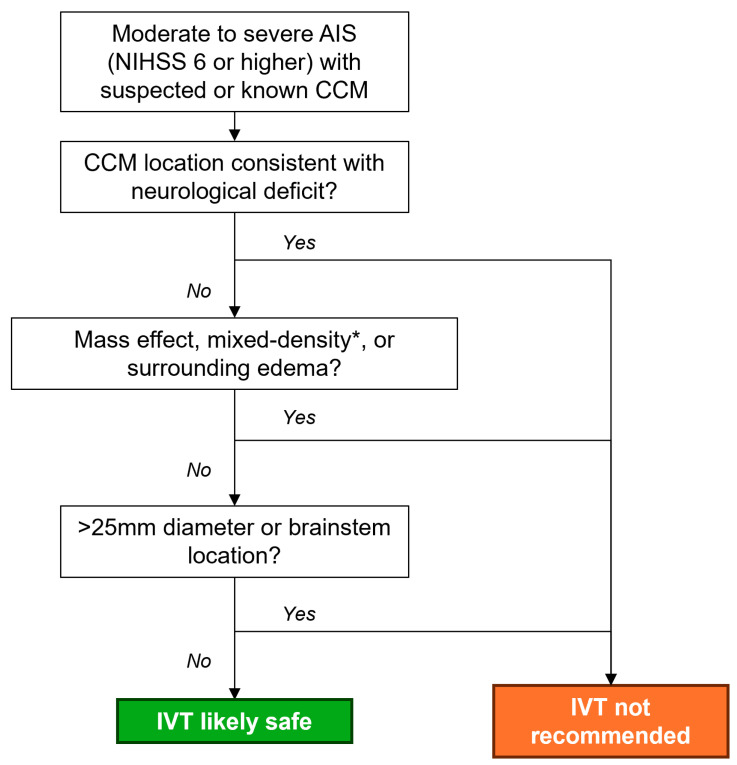
Proposed intravenous thrombolysis (IVT) decision flow chart. * Mixed density refers to variably aged blood products; calcifications are a common feature of CCMs are should not be considered a contraindication to IVT. Abbreviations: AIS—acute ischemic stroke, CCM—cerebral cavernous malformations.

While our data suggest that IVT may be associated with better outcomes for many AIS patients with CCMs, it is important to acknowledge the clinical challenges inherent to determining IVT appropriateness during AIS triage. First, acute CCM rupture or seizures related to CCM lesions may present as AIS mimics; therefore, treatment with IVT should be reserved for patients whose symptoms do not localize to the CCM lesion. Furthermore, the gold-standard test for diagnosis and characterization of CCMs is magnetic resonance imaging, which is not routinely used during acute stroke triage in most clinical facilities worldwide [25,26,27]. On computed tomography, unruptured CCMs can be distinguished from parenchymal hemorrhages or previously ruptured CCMs by a homogenous hyperdense appearance with a lack of mass effect or surrounding edema [28]; IVT should only be considered in these cases. Prior studies have shown that increased CCM size, with a diameter greater than 25 mm [29], and brainstem location pose a particularly high risk of rupture, which can be neurologically devastating and life-threatening [2]; thus, IVT should be withheld from patients with large CCMs or lesions in the brainstem. Based on these considerations, we propose a decision flow-chart to aid clinicians in IVT decision-making for patients with suspected CCMs (Figure 2). Future prospective trials are needed to confirm the safety and efficacy of IVT for patients with known or suspected CCM lesions.

Several limitations inherent to administrative database studies must be acknowledged. First, the identification of CCMs depended on clinical recognition and appropriate diagnostic coding, which may exclude undiagnosed or asymptomatic lesions not captured without magnetic resonance imaging. This could introduce a selection bias toward patients with preexisting neurological deficits from a known CCM, although this bias would likely affect both treatment groups similarly and should not confound comparative analyses. Moreover, patients who experience hemorrhagic complications may be more likely to undergo detailed neuroimaging, potentially inflating CCM detection in this subgroup. However, the similar hemorrhage rates observed between groups mitigate concerns regarding this source of bias. Second, the dataset also lacked information on CCM-specific characteristics such as lesion size, multiplicity, association with developmental venous anomalies, and surrounding edema, as the impact of these features on thrombolysis safety remains uncertain. The NRD also does not provide information on treatment time windows for thrombolytic agents, though this is not expected to bias outcomes as the non-treatment group would not be affected; nevertheless, IVT may be preferentially used in clinical practice for CCM patients with earlier time windows compared to non-CCM populations, which could impact the generalizability of our findings to all otherwise eligible AIS patients with CCM. Other potentially relevant information that is lacking in the NRD include type of IVT (alteplase, Tenecteplase, etc.) and laboratory or physiological values (e.g., hemoglobin, glucose, blood pressure, coagulation metrics, etc.). Finally, while the NRD provides information on discharge destination which can be used as a surrogate marker for functional status, formal functional assessments at the time of discharge or at later time points (e.g., 90 days or 1 year) are not available. Future studies are needed to further validate our findings with more commonly used functional outcome markers such as the modified Rankin scale [30].

## 5. Conclusions

In our comprehensive review of four major medical literature databases, only 34 cases of IVT administration for AIS patients with CCMs were identified, highlighting a severe scarcity of clinical data on the safety and efficacy of IVT in this unique population. A retrospective analysis of a nationwide cohort of 846 CCM patients hospitalized for AIS was conducted, and we found that, compared to no IVT treatment, IVT was associated with a three-fold increase in the odds of functional independence without increased risk of in-hospital mortality or hemorrhagic complications. These findings suggest that thrombolysis may be reasonably considered for select CCM patients presenting with AIS, pending confirmation in future prospective studies.

## Figures and Tables

**Table 1:** Literature review of intravenous thrombolysis treatment for acute ischemic stroke patients with cerebral cavernous malformations.

Study	Setting	No. of Patients	No. of IVT-Treated CCM Patients	No. of IVT-Treated CCM Patients with sICH (%)
Erdur et al. (2014) [19]	Single Center, Germany	350	9	1 (11.1%)
Schwarzbach et al. (2018) [14]	Single Center, Germany	34	13	0 (0.0%)
Lin et al. (2023) [15]	Multicenter, China	5	5	0 (0.0%)
Stone et al. (2019) [13]	Single Center, USA	56	4	0 (0.0%)
Gattringer et al. (2013) [18]	Single Center, Austria	1	1	1 (100.0%)
Henninger et al. (2010) [17]	Single Center, USA	1	1	0 (0.0%)
Kargiotis et al. (2019) [16]	Single Center, Greece	1	1	0 (0.0%)
Total	-	-	34	2 (5.9%)

**Table 2:** Patient characteristics of CCM patients hospitalized for AIS in the NRD from 2016 to 2022.

	Total Cohort	No IVT	IVT	
Characteristics—Median (Q1–Q3) and % (*n*)	*N* = 846	*N* = 606	*N* = 240	*p*-Value
Age (years)	71 (60–79)	72 (61–80)	69 (56–77)	0.021
Female sex	46.8% (396)	45.3% (274)	50.6% (121)	0.28
Urban teaching hospital	85.8% (725)	84.3% (511)	89.6% (215)	0.16
NIH stroke scale	9 (6–16)	9 (6–16)	9 (6–17)	0.31
Additional acute treatment				
Endovascular thrombectomy	17.4% (147)	16.2% (98)	20.4% (49)	0.31
Endovascular angioplasty	4.2% (35)	4.8% (29)	2.6% (6)	0.34
Chronic medications				
Anticoagulant	8.1% (68)	9.9% (60)	3.6% (9)	0.010
Antiplatelet	9.1% (77)	10.0% (61)	6.7% (16)	0.29
Comorbidities				
Hypertension	84.0% (711)	85.4% (517)	80.7% (193)	0.26
Atrial fibrillation	23.9% (202)	23.3% (141)	25.5% (61)	0.63
Diabetes	34.2% (289)	35.5% (215)	30.9% (74)	0.28
Smoking	33.5% (283)	31.8% (193)	37.8% (91)	0.28
Hyperlipidemia	59.6% (504)	60.2% (365)	58.1% (139)	0.68
Dementia	10.7% (91)	12.0% (73)	7.5% (18)	0.20
Cervical artery dissection	3.7% (31)	3.9% (24)	3.1% (8)	0.72
Intracranial atherosclerosis	10.1% (85)	10.8% (65)	8.4% (20)	0.42
Peripheral artery disease	3.3% (28)	3.0% (18)	4.1% (10)	0.53
Myocardial infarction	2.8% (23)	2.8% (17)	2.6% (6)	0.89
Ischemic heart disease	19.9% (168)	20.7% (126)	17.8% (43)	0.50
Chronic kidney disease	10.4% (88)	11.9% (72)	6.6% (16)	0.081
Liver disease	2.0% (17)	2.0% (12)	2.0% (5)	0.99
Coagulopathy	8.4% (71)	9.4% (57)	6.0% (14)	0.25
Congestive heart failure	16.2% (137)	16.7% (101)	14.7% (35)	0.56
Mood disorder	14.4% (122)	14.0% (85)	15.4% (37)	0.74
Anxiety disorder	13.5% (114)	11.5% (70)	18.6% (45)	0.055
Headache disorder	6.3% (53)	4.5% (27)	10.9% (26)	0.012
Elixhauser comorbidity index	12 (7–18)	12 (7–18)	12 (7–18)	0.78

**Table 3:** Study outcomes of IVT versus no IVT for AIS patients with CCM.

	Unadjusted Comparisons			Adjusted Comparisons	
Outcomes	No IVT (*n* = 606)	IVT (*n* = 240)	*p*-Value	Estimand [95% CI]	*p*-Value
Functional Independence	24.6% (149)	46.4% (111)	<0.001	3.04 [1.98–4.68]	<0.001
Death	8.3% (50)	8.5% (20)	0.96	1.40 [0.52–3.76]	0.50
Intracranial hemorrhage	16.1% (97)	20.3% (49)	0.30	1.01 [0.53–1.93]	0.97
Subarachnoid hemorrhage	3.3% (20)	3.5% (8)	0.93	1.26 [0.38–4.52]	0.72
Intraparenchymal hemorrhage	13.3% (80)	18.2% (44)	0.22	1.14 [0.55–2.38]	0.73
Length of hospital stay (days)	6 (3–14)	4 (3–9)	0.014	0.65 [0.53–0.81]	<0.001

Note: Unadjusted outcomes are presented as % (*n*) or median (Q1–Q3); adjusted estimands for functional independence, death, intracranial hemorrhage, subarachnoid hemorrhage, and intraparenchymal hemorrhage were odds ratios of IVT versus no IVT, and the estimand for length of stay was Poisson rate ratio of IVT versus no IVT. Multivariable adjustments included all captured variables presented in Table 1. *p*-values less than 0.05 were deemed statistically significant.

**Table 4:** Study outcomes among patients without prior anticoagulant use or coagulopathy.

	Unadjusted Comparisons			Adjusted Comparisons	
Outcomes	No IVT (*n* = 546)	IVT (*n* = 231)	*p*-Value	Estimand [95% CI]	*p*-Value
Functional Independence	23.1% (126)	45.9% (106)	<0.001	3.04 [1.86–4.99]	<0.001
Death	8.0% (44)	8.1% (19)	0.98	1.36 [0.46–4.05]	0.58
Intracranial hemorrhage	15.9% (87)	19.8% (46)	0.37	0.92 [0.46–1.83]	0.80
Subarachnoid hemorrhage	3.1% (17)	3.0% (7)	0.93	1.09 [0.21–5.67]	0.92
Intraparenchymal hemorrhage	13.4% (73)	17.6% (41)	0.32	0.98 [0.46–2.08]	0.95
Length of hospital stay	6 (3–13)	4 (3–10)	0.049	0.67 [0.53–0.84]	<0.001

Note: Unadjusted outcomes are presented as % (*n*) or median (Q1–Q3); adjusted estimands for functional independence, death, intracranial hemorrhage, subarachnoid hemorrhage, and intraparenchymal hemorrhage were odds ratios of IVT versus no IVT, and the estimand for length of stay was Poisson rate ratio of IVT versus no IVT. Multivariable adjustments included all captured variables presented in Table 1. *p*-values less than 0.05 were deemed statistically significant.

## Data Availability

All data used in this study are publicly available for purchase at https://hcup-us.ahrq.gov/.

## Supplementary Materials

The following supporting information can be downloaded at: https://www.mdpi.com/article/10.3390/neurosci7010024/s1, Table S1: Search Strategy; Table S2: ICD-10 codes used in this study; Table S3: Study outcomes among patients who did not undergo endovascular thrombectomy.
