# Supplementary material for: Safety and Efficacy of Stroke Thrombolysis for Patients with Cerebral Cavernous Malformations: Literature Review and Nationwide Cohort Study
Source: NeuroSci. 2026 Feb 8;7(1):24. doi: 10.3390/neurosci7010024 (PMC12921825; doi:10.3390/neurosci7010024)
Supplement: Supplementary file 1 [file neurosci-07-00024-s001.zip › neurosci-3944808-supplementary.pdf]

NeuroSci

MDPI

Table S1. Search Strategy.

Database	Search Strategy.
PubMed	(stroke[MeSH Terms] OR stroke[Title/Abstract] OR infarct*[Title/Abstract] OR ischemia[Title/Abstract] OR ischaemia[Title/Abstract] OR "cerebral infarction"[MeSH Terms]) AND (thrombolysis[Title/Abstract] OR "thrombolytic therapy"[MeSH Terms] OR alteplase[Title/Abstract] OR tenecteplase[Title/Abstract] OR thrombolytic*[Title/Abstract] OR tPA[Title/Abstract] OR "tissue plasminogen activator"[Title/Abstract] OR urokinase[Title/Abstract] OR "fibrinolytic agents"[MeSH Terms]) AND ("cerebral cavernous malformation*"[Title/Abstract] OR cavernoma*[Title/Abstract] OR "cavernous angioma*"[Title/Abstract] OR "cavernous hemangioma*"[Title/Abstract] OR "hemangioma, cavern-ous, central nervous system"[MeSH Terms])
Embase (Elsevier)	('cerebrovascular accident'/exp OR stroke:ab,ti OR infarct*:ab,ti OR ischemia:ab,ti OR ischaemia:ab,ti OR 'brain infarction'/exp) AND ('fibrinolytic therapy'/exp OR thrombolysis:ab,ti OR 'alteplase'/exp OR alteplase:ab,ti OR 'tenecteplase'/exp OR tenecteplase:ab,ti OR thrombolytic*:ab,ti OR tpa:ab,ti OR 'tissue plasminogen activator':ab,ti OR 'urokinase'/exp OR urokinase:ab,ti) AND ('cavernous hemangioma'/exp OR 'cerebral cavernous malformation*':ab,ti OR cavernoma*:ab,ti OR 'cavernous angioma*':ab,ti OR 'cavernous hemangioma*':ab,ti)
Web of Science	TS=((stroke OR infarct* OR ischemia OR ischaemia OR "cerebral infarction") AND (thrombolysis OR alteplase OR tenecteplase OR thrombolytic* OR tPA OR "tissue plasminogen activator" OR urokinase OR "fibrinolytic therapy") AND ("cerebral cavernous malformation*" OR cavernoma* OR "cavernous angioma*" OR "cavernous hemangioma*"))
Scopus	TITLE-ABS-KEY((stroke OR infarct* OR ischemia OR ischaemia OR "cerebral infarction") AND (thrombolysis OR alteplase OR tenecteplase OR thrombolytic* OR tPA OR "tissue plasminogen activator" OR urokinase OR "fibrinolytic therapy") AND ("cerebral cavernous malformation*" OR cavernoma* OR "cavernous angioma*" OR "cavernous hemangioma*"))

Table S2. ICD-10 codes used in this study.

Variable	Code(s)
**Inclusion**	
Stroke (top dx code)	G463, G464, G465, G466, G467, I6300, I63011, I63012, I63013, I63019, I6302, I63031, I63032, I63033, I63039, I6309, I6310, I63111, I63112, I63113, I63119, I6312, I63131, I63132, I63133, I63139, I6319, I6320, I63211, I63212, I63213, I63219, I6322, I63231, I63232, I63233, I63239, I6329, I6330, I63311, I63312, I63313, I63319, I63321, I63322, I63323, I63329, I63331, I63332, I63333, I63339, I63341, I63342, I63343, I63349, I6339, I6340, I63411, I63412, I63413, I63419, I63421, I63422, I63423, I63429, I63431, I63432, I63433, I63439, I63441, I63442, I63443, I63449, I6349, I6350, I63511, I63512, I63513, I63519, I63521, I63522, I63523, I63529, I63531, I63532, I63533, I63539, I63541, I63542, I63543, I63549, I6359, I636, I6381, I6389, I639
NIHSS 5 or greater	R297
CCM	Q283, D18.02
IVT	3E03317,Z9282
**Exclusion**	
Vasculitis/arteritis	I677, M31
Endocarditis	I33
Cerebral amyloid angiopathy	I680
Meningioma	D32
Moyamoya disease	I675
**Treatments**	
Endovascular thrombectomy	03CG3, 03CK3, 03CL3, 03CP3, 03CQ3
Endovascular angioplasty	037G3 ,037H3, 037J3, 037K3, 037L3, 037P3, 037Q3
**Comorbidities**	
Afib	I480,I4811,I4819,I4820,I4821,I483,I484,I4891,I4892
Chronic kidney disease	N18
Ischemic heart disease	I25, I21, I22
Peripheral artery disease	I70, K550, K551
Dementia	F00. F01. F02. F03. G30, I6991
Intracranial atherosclerosis	I672

Hyperlipidemia	E7800, E7801, E781,E782,E783,E7841,E7849,E785
Smoking	Z720,Z87891,F17210
Anticoagulant use	D6832,Z7901
Antiplatelet use	Z7902
Chronic kidney disease	N18
Headache disorder	G44, G43, R51
Dissection	I7771, I7774,I7775
Anxiety disorder	F40, F41, F42, F43, F44, F45, F48
Mood disorders	F30, F31, F32, F33, F34, F39,

All other comorbidities within Elixhauser and Charleston comorbidity indices are extracted using "icd" package (v 4.0.9) in R.

Table S3. Study outcomes among patients who did not undergo endovascular thrombectomy.

	**Unadjusted Comparisons**			**Adjusted Comparisons**	
Outcomes	No IVT (n=508)	IVT (n=191)	p-value	Estimand [95%CI]	p-value
Functional Independence	25.4% (129)	47.7% (91)	<0.001	2.93 [1.81-4.75]	<0.001
Death	6.9% (35)	8.1% (15)	0.70	1.74 [0.58-5.20]	0.32
Intracranial hemorrhage	13.5% (69)	15.1% (29)	0.72	1.00 [0.44-2.24]	0.99
Subarachnoid hemorrhage	1.4% (7)	2.8% (5)	0.42	2.53 [0.35-18.3]	0.35
Intraparenchymal hemorrhage	12.4% (63)	14.1% (27)	0.69	1.02 [0.44-2.37]	0.97
Length of hospital stay	6 (3-14)	4 (2-8)	0.005	0.60 [0.47-0.77]	<0.001

Note: Unadjusted outcomes are presented as % (n) or median (Q1-Q3); adjusted estimands for functional inde-pendence, death, intracranial hemorrhage, subarachnoid hemorrhage, and intraparenchymal hemorrhage were odds ratios of IVT versus no IVT, and the estimand for length of stay was Poisson rate ratio of IVT versus no IVT. Multivariable adjustments included all captured variables presented in Table 1. P-values less than 0.05 were deemed statistically significant.
